# Vincristine Sulfate Liposome Injection with Bendamustine and Rituximab as First-Line Therapy for B-Cell Lymphomas: A Phase I Study

**DOI:** 10.1093/oncolo/oyab079

**Published:** 2022-03-07

**Authors:** Thomas Ollila, James Butera, Pamela Egan, John Reagan, Anthony Thomas, Inna Yakirevich, Kelsey MacKinnon, Jeannine Margolis, Jessica McMahon, Valerie Rosati, Adam J Olszewski

**Affiliations:** Department of Medicine, Alpert Medical School of Brown University, Providence, RI, USA; Division of Hematology-Oncology, Rhode Island Hospital, Providence, RI, USA; Department of Medicine, Alpert Medical School of Brown University, Providence, RI, USA; Division of Hematology-Oncology, Rhode Island Hospital, Providence, RI, USA; Department of Medicine, Alpert Medical School of Brown University, Providence, RI, USA; Division of Hematology-Oncology, Rhode Island Hospital, Providence, RI, USA; Department of Medicine, Alpert Medical School of Brown University, Providence, RI, USA; Division of Hematology-Oncology, Rhode Island Hospital, Providence, RI, USA; Department of Medicine, Alpert Medical School of Brown University, Providence, RI, USA; Division of Hematology-Oncology, Rhode Island Hospital, Providence, RI, USA; Division of Hematology-Oncology, Rhode Island Hospital, Providence, RI, USA; Brown University Oncology Research Group (BrUOG), Providence, RI, USA; Brown University Oncology Research Group (BrUOG), Providence, RI, USA; Lifespan Oncology Clinical Research, Providence, RI, USA; Lifespan Oncology Clinical Research, Providence, RI, USA; Department of Medicine, Alpert Medical School of Brown University, Providence, RI, USA; Division of Hematology-Oncology, Rhode Island Hospital, Providence, RI, USA

**Keywords:** vincristine sulfate liposome injection, bendamustine, rituximab, B-cell lymphoma, phase I

## Abstract

**Background:**

We conducted an investigator-initiated, phase I trial of vincristine sulfate liposomal injection (VSLI) in combination with bendamustine and rituximab (BR) for indolent B-cell (BCL) or mantle cell lymphoma.

**Methods:**

Participants received 6 cycles of standard BR with VSLI at patient-specific dose determined by the Escalation with Overdose Control (EWOC) model targeting 33% probability of dose-limiting toxicity (DLT). Maximum tolerated dose (MTD) was the primary endpoint; secondary endpoints included rates of adverse events (AEs), overall response rate (ORR), and complete response (CR). Vincristine sulfate liposomal injection is FDA approved for the treatment of patients with recurrent Philadelphia chromosome-negative (Ph−) acute lymphoblastic leukemia (ALL).

**Results:**

Among 10 enrolled patients, VSLI was escalated from 1.80 to 2.24 mg/m^2^, with one DLT (ileus) at 2.04 mg/m^2^. Two patients discontinued VSLI early. The most common AE included lymphopenia (100%), constipation, nausea, infusion reaction (each 60%), neutropenia, and peripheral neuropathy (50%). Grade 3/4 AE included lymphopenia (90%), neutropenia (20%), and ileus (10%), with prolonged grade ≥2 lymphopenia observed in most patients. Calculated MTD for VSLI was 2.25 mg/m^2^ (95% Bayesian credible interval: 2.00-2.40). Overall response was 100% with 50% CR. With median follow-up 26 months, 4/10 patients experienced recurrence and 1 died.

**Conclusion:**

Vincristine sulfate liposomal injection at 2.25 mg/m^2^ can be safely combined with BR for indolent B-cell lymphoma, but given observed toxicities and recurrences, we did not pursue an expanded cohort.

Clinical Trials Registration Number: ClinicalTrials.gov identifier NCT02257242.

## Teaser

This article reports the results of a phase I trial of vincristine sulfate liposomal injection in combination with bendamustine and rituximab for indolent B-cell or mantle cell lymphoma.

**Lessons Learned**

- Vincristine sulfate liposomal injection (VSLI) can be safely combined with full doses of bendamustine and rituximab (BR) for first-line treatment of indolent B-cell lymphoma (BCL) and mantle cell lymphoma (MCL).
- The use of EWOC model-based design allowed for dose escalation in a small cohort, although a larger study would be needed to further characterize the toxicity and efficacy of BR plus VSLI.
- The observed rates of prolonged lymphopenia, peripheral neuropathy, and lymphoma recurrences suggest that approaches other than intensifying chemotherapy may provide more benefit for patients with BCL/MCL.

## Discussion

**Figure 1.**
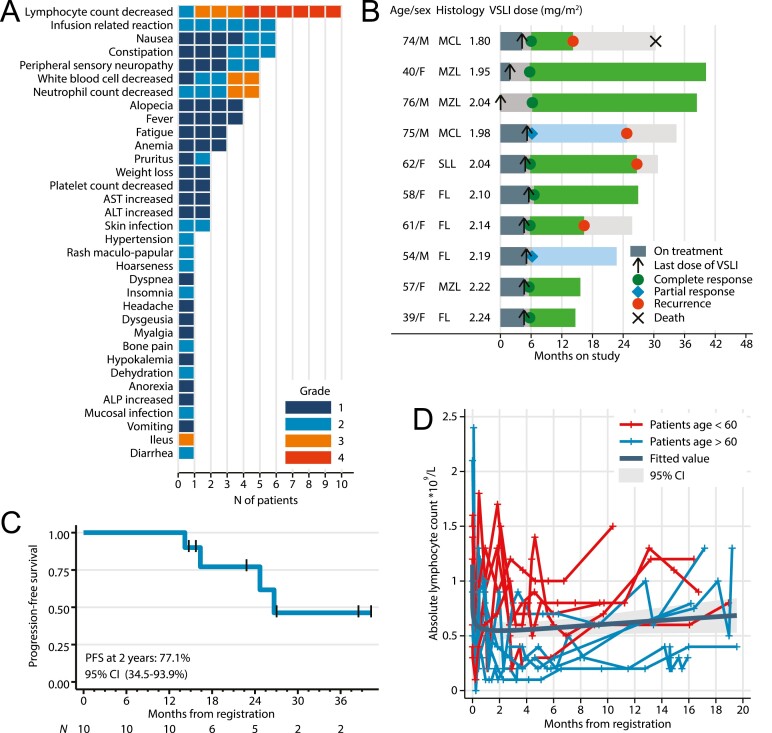
(**A**) AE by grade, (**B**) swimmer’s plot indicating each patient‘s age, sex, histology, dose of VSLI, response at the end of therapy and disposition; (**C**) estimate of progression-free survival (PFS); (**D**) absolute lymphocyte counts during and after therapy, including a mean estimate (fractional polynomial fit) with 95% confidence interval. Abbreviations: AST, aspartate transaminase; ALT, alanine transaminase; ALP, alkaline phosphatase; CI, confidence interval; FL, follicular lymphoma; MCL, mantle cell lymphoma; MZL, marginal zone lymphoma; SLL, small lymphocytic lymphoma; VSLI, vincristine sulfate liposome injection.

Bendamustine and rituximab is a common regimen for treatment of indolent B-cell lymphoma and mantle cell lymphoma (MCL) and has non-overlapping toxicities with vincristine [Lancet 2013;381:1203-1210]. We hypothesized that VSLI, a liposome-encapsulated formulation of vincristine with an improved therapeutic window, could be safely combined with BR, potentially improving its efficacy [J Clin Oncol 2013;31:676-683].In this open-label phase I Brown University Oncology Research Group trial, we combined BR (bendamustine 90 mg/m^2^ on days 1-2, rituximab 375 mg/m^2^ on day 1, every 28 days) with VSLI administered on day 2. The dose of VSLI was calculated for each enrolled patient (without any intra-patient escalation), starting from 1.8 mg/m^2^ using the EWOC Bayesian model which used data on the presence or absence of DLT in all previously treated subjects and targeted 33% probability of DLT [Stat Sci 2010;25:217-226] . Simulations indicated that 10 patients would be sufficient to determine the MTD defined as median of the 95% credible interval (CI). Dose-limiting toxicity was determined during cycle 1 as grade (G)4 neutropenia lasting >7 days (or G3 with fever/infection), G4 thrombocytopenia (or G3 requiring transfusion), or any G3/G4 non-hematologic toxicity. Among 10 treated patients with indolent B-cell or mantle cell lymphoma, we did not observe any unexpected toxicities (Fig. 1A and B). G3/4 AE were limited to lymphopenia (90%), neutropenia (20%), and 1 DLT (G3 ileus which occurred on day 15 of cycle 1). The MTD of VSLI was calculated at 2.25 mg/m^2^ (95%CI, 2.00-2.40). Two patients discontinued VSLI early (1 for DLT in cycle 1, and 1 for G2 neuropathy in cycle 3), but all completed 6 cycles of BR. The rates of ORR (100%) and CR (50%), assessed using the 2007 International Working Group criteria, appeared promising in comparison with historical BR, but we subsequently observed recurrent lymphoma in 4 of 10 patients. One patient with recurrent blastoid MCL died, while others remain alive. With a 2-year PFS estimate of 77% (Fig. 1C), the BR+VSLI combination was deemed not sufficiently promising to warrant an expanded cohort. This assessment was further supported by prolonged lymphopenia, particularly in patients older than 60 years (Fig. 1D). Five of 8 patients (63%) followed for >12 months continued to have *G* ≥ 2 lymphopenia (lymphocyte count <0.8 × 10^9^/L). CD4 T-cell counts obtained in 5 patients at 18-24 months after treatment ranged from 0.19 to 0.40 × 10^9^/L, although we recorded no opportunistic infections. Our study suggests that BR, a regimen with relatively low acute toxicity, can be intensified by addition of VSLI, a drug with non-overlapping AE profile. However, considering potential for prolonged immunosuppression (also observed in clinical trials of BR with maintenance rituximab), concerns about serious complications in case of COVID-19 infection, and emergence of highly active novel immunotherapies, the investigators concluded that patients with B-cell and mantle cell lymphoma might benefit from more innovative therapeutic approaches.

## Trial Information

**Table:** 

Disease	Lymphoma—non-Hodgkin
Stage of disease/treatment	Primary
Prior therapy	No designated number of regimens
Type of study	Phase I, accelerated titration
Primary endpoint	Maximum tolerated dose
Secondary endpoints	Toxicity, deliverability, tolerability
Investigator’s analysis	Active but results overtaken by other developments

## Additional Details of Endpoints or Study Design

### Patients

Patients provided a written informed consent prior to participating in the study. Eligibility criteria included: CD20 positive, histologically confirmed BCL of the following subtypes: follicular, mantle cell, marginal zone, small lymphocytic, lymphoplasmacytic, or indolent B-cell lymphoma not otherwise specified; radiological measurable disease (defined as any measurable lesion >1.5 cm on the staging CT scan); previous treatment for the lymphoma was allowed except as in exclusion criteria; age ≥18 years, ECOG performance status 0 or 1; life expectancy >6 months; adequate organ and bone marrow function (including absolute neutrophil count ≥10 × 10^9^/L, ≥75 × 10^9^/L, total bilirubin within normal limits, aspartate and alanine transaminase <2.5 × institutional upper limit of normal, and creatinine clearance ≥50 mL/min/1.73 m^2^; adequate contraception. Exclusion criteria were: history of any allergies to study drugs; any lymphoma-directed therapy within 4 weeks prior to registration, or with residual AE >G1 from prior therapy; any prior treatment with VSLI; prior treatment with bendamustine or vincristine within 180 days of enrollment; use of any other investigational or anti-cancer therapy; known central nervous system involvement by the lymphoma; active peripheral sensory or motor neuropathy >G1; history of demyelinating condition; documented positive test for human anti-chimeric antibody; treatment with strong inhibitors or inducers of CYP3A; uncontrolled intercurrent illness; prisoner status; pregnancy or breast-feeding status; known HIV or active hepatitis B infection; any prior or active cancer precluding safe participation.

### Study Treatment

Patients received bendamustine 90 mg/m^2^ on days 1 and 2, rituximab 375 mg/m^2^ on day 2, and VSLI (at patient-specific dose) on day 2 of each 28-day cycle. Treatment was continued for 6 cycles in the absence of progression. Patients >65 years old were required to receive pegfilgrastim with each cycle. Intrapatient dose escalation was not permitted. The dose of VSLI was escalated in cohorts of *n* = 1 and calculated for each patient using the Escalation with Overdose Control (EWOC) Bayesian model targeting the probability of DLT of 33%, with potential VSLI doses ranging from 1.8 up to 2.3 mg/m^2^. The sample size of *n* = 10 was based on simulation of 500 trials with true MTD of VSLI ranging between 1.8 and 2.2 mg/m^2^.

### Objectives and Endpoints

The primary objective was to establish the maximum tolerated dose (MTD) and dose-limiting toxicity (DLT) of VSLI in combination with BR, and the relevant DLT. The secondary objectives were to evaluate cumulative rates of toxicities of the BR+VSLI combination, to evaluate tolerability assessed by the rate of completion of the planned 6 cycles of chemotherapy, and to evaluate overall response rate (ORR) and complete response (CR) rate to BR+VSLI in the study cohort at the end of six cycles of therapy. The MTD was defined as median of the Bayesian 95% credible interval (CI) for the dose resulting in 33% predicted rate of DLT, as calculated by the EWOC model using data from all 10 subjects. Dose-limiting toxicity was determined during cycle 1 and defined as grade (G) 4 neutropenia lasting >7 days (or G3 with fever/ infection), G4 thrombocytopenia (or G3 requiring transfusion), or any G3/G4 nonhematologic toxicity. Responses were assessed using the standard computed tomography (CT)-based International Working Group criteria for Malignant Lymphoma.

## Drug Information

**Table:** 

Bendamustine	
Generic/working name	Bendamustine
Trade name	Treanda, Bendeka
Company name	Cephalon, Inc.; Teva Pharmaceutical Industries Ltd.
Drug type	Cytotoxic
Drug class	Alkylating agent
Dose	90 mg/m²
Route	i.v.
Schedule of administration	Administered on days 1 and 2 of each cycle, 28 days

Rituximab	
Generic/working name	Rituximab
Trade name	Rituxan
Company name	Genentech
Drug type	Antibody
Drug class	CD20
Dose	375 mg/m²
Route	i.v.
Schedule of administration	Administered on day 1 of each cycle, every 28 days

Vincristine sulfate liposome injection	
Generic/working name	Vincristine sulfate liposome injection
Trade name	Marqibo
Company name	Acrotech Biopharma
Drug type	Liposomal encapsulated
Drug class	Microtubule-targeting agent
Dose	1.8-2.4 mg/m²
Route	i.v.
Schedule of administration	Administered on day 2 of each cycle, after bendamustine, every 28 days. Patient-specific dose determined by the EWOC model

## Dose Escalation Table

**Table:** 

Dose level	Dose of drug: bendamustine	Dose of drug: rituximab	Dose of drug: vincristine sulfate liposome injection	Number enrolled	Number evaluable for toxicity
1	90	375	1.80	1	1
2	90	375	1.95	1	1
3	90	375	2.04	1	1
4	90	375	1.98	1	1
5	90	375	2.04	1	1
6	90	375	2.10	1	1
7	90	375	2.14	1	1
8	90	375	2.19	1	1
9	90	375	2.22	1	1
10	90	375	2.24	1	1

### Patient Characteristics

**Table:** 

Number of patients, male	4
Number of patients, female	6
Stage	Stage 2: *n* = 1, stage 3: *n* = 1, stage 4: *n* = 8
Age	Median (range): 59.5 (39-76) years
Number of prior systemic therapies	Median (range):0 (0-0)
Performance status: ECOG	0—8 1—2 2—0 3—0 Unknown—0
Cancer types or histologic subtypes	Follicular lymphoma, 4 Marginal zone lymphoma, 3 Mantle cell lymphoma, 2 Small lymphocytic lymphoma, 1

### Primary Assessment Method

**Table:** 

Title	Maximum tolerated dose
Number of patients screened	12
Number of patients enrolled	10
Number of patients evaluable for toxicity	10
Number of patients evaluated for efficacy	10
Evaluation method	MTD was defined as median of the Bayesian 95% credible interval (CI) calculated from the model. Response rates were assessed using the standard computed tomography (CT)-based International Working Group criteria for Malignant Lymphoma
Response assessment CR	*n* = 8 (80%)
Response assessment PR	*n* = 2 (20%)
Response assessment SD	*n* = 0 (0%)
Response assessment PD	*n* = 0 (0%)
(Median) duration assessments PFS	27 months, CI: 14 to not reached

#### Outcome Notes

The calculated MTD for VSLI was 2.25 mg/m^2^ (95% Bayesian credible interval: 2.00-2.40).

## Adverse Events, All Dose Levels, All Cycles

**Table:** 

Name	∗NC/NA	1	2	3	4	5	All grades
Lymphocyte count decreased	0%	0%	10%	30%	60%	0%	100%
Cytokine release syndrome/acute infusion reaction	40%	0%	60%	0%	0%	0%	60%
Nausea	40%	40%	20%	0%	0%	0%	60%
Constipation	40%	30%	30%	0%	0%	0%	60%
Neutrophil count decreased	50%	0%	30%	20%	0%	0%	50%
White blood cell decreased	50%	10%	20%	20%	0%	0%	50%
Peripheral sensory neuropathy	50%	30%	20%	0%	0%	0%	50%
Fever	60%	40%	0%	0%	0%	0%	40%
Alopecia	60%	40%	0%	0%	0%	0%	40%
Anemia	70%	30%	0%	0%	0%	0%	30%
Fatigue	70%	30%	0%	0%	0%	0%	30%
Skin infection	80%	0%	20%	0%	0%	0%	20%
ALT, SGPT (serum glutamic pyruvic transaminase)	80%	20%	0%	0%	0%	0%	20%
AST, SGOT(serum glutamic oxaloacetic transaminase)	80%	20%	0%	0%	0%	0%	20%
Platelet count decreased	80%	20%	0%	0%	0%	0%	20%
Weight loss	80%	20%	0%	0%	0%	0%	20%
Pruritus	80%	10%	10%	0%	0%	0%	20%
Diarrhea	90%	0%	10%	0%	0%	0%	10%
Ileus	90%	0%	0%	10%	0%	0%	10%
Vomiting	90%	10%	0%	0%	0%	0%	10%
Mucosal infection	90%	0%	10%	0%	0%	0%	10%
Alkaline phosphatase increased	90%	10%	0%	0%	0%	0%	10%
Anorexia	90%	10%	0%	0%	0%	0%	10%
Dehydration	90%	0%	10%	0%	0%	0%	10%
Hypokalemia	90%	10%	0%	0%	0%	0%	10%
Bone pain	90%	0%	10%	0%	0%	0%	10%
Myalgia	90%	10%	0%	0%	0%	0%	10%
Dysgeusia	90%	10%	0%	0%	0%	0%	10%
Headache	90%	10%	0%	0%	0%	0%	10%
Insomnia	90%	0%	10%	0%	0%	0%	10%
Dyspnea	90%	10%	0%	0%	0%	0%	10%
Voice changes/dysarthria (eg, hoarseness, loss or alteration in voice, laryngitis)	90%	0%	10%	0%	0%	0%	10%
Rash maculo-papular	90%	0%	10%	0%	0%	0%	10%
Hypertension	90%	0%	10%	0%	0%	0%	10%

One patient experienced a serious AE with ileus (DLT), fever, and constipation on day 13 of cycle 1. One patient experienced a catheter-associated thrombosis on day 8 of cycle 1. Both patients recovered and continued their therapy without interruption or delay.

## Serious Adverse Events

**Table:** 

Name	Grade	Attribution
Ileus	3	Probable
Constipation	2	Probable
Fever	1	Possible
Thromboembolic event	2	Unrelated

## Dose-Limiting Toxicities

**Table:** 

	Dose of drug: bendamustine	Dose of drug: rituximab	Dose of drug: vincristine sulfate liposome injection	Number enrolled	Number evaluable for toxicity	Number with a dose limiting toxicity	Dose limiting toxicity information
	90	375	2.04	1	1	1	Ileus

## Assessment, Analysis, and Discussion

**Table:** 

Completion	Study completed
Investigator’s Assessment	Active but results overtaken by other developments

BR is the most frequent immunochemotherapy regimen for first-line treatment of indolent B-cell lymphomas (BCL, including grade 1-3a follicular lymphoma [FL], marginal zone lymphoma [MZL], small lymphocytic lymphoma [SLL], and lymphoplasmacytic lymphoma) or for older patients with mantle cell lymphoma (MCL) [4, 5]. In clinical trials, BR has shown improved PFS over cyclophosphamide-based regimens, as well as better toxicity profile [1, 6]. Vincristine, a microtubule-targeting alkaloid, is one of the most active agents against B-cell lymphomas, and has non-overlapping mechanism of action and toxicity profile with BR, suggesting a value of potential combination. However, in a prior trial, the combination of bendamustine with vincristine and prednisone resulted in high rates of thrombocytopenia and peripheral neuropathy and was not pursued further [7].

Vincristine sulfate liposomal injection is a formulation of vincristine encapsulated in sphingomyelin/cholesterol-containing liposomes, designed to improve its therapeutic index and tumor drug exposure [8, 9]. Vincristine sulfate liposomal injection at an uncapped dose of 2.25 mg/m^2^/week is currently approved for the treatment of adult patients with Philadelphia chromosome-negative acute lymphoblastic leukemia in second or greater relapse, or whose disease has progressed following 2 or more anti-leukemia therapies [2]. Vincristine sulfate liposomal injection (2.0 mg/m^2^) was previously substituted for vincristine in a combination with cyclophosphamide, doxorubicin, and prednisone with or without rituximab in a phase II trial, and no excessive toxicity was reported [10]. In a pivotal phase II trial, VSLI was tolerable and had modest single-agent activity (ORR 25%) in relapsed or refractory non-Hodgkin lymphomas at a dose of 2.0 mg/m^2^ every 2 weeks [11]. We sought to determine the MTD and DLT of VSLI administered every 28 days in combination with BR. We hypothesized that VSLI and BR can be safely combined, possibly improving on the activity of BR alone. The BR+VSLI combination could be further studied in indolent BCL, as well as potentially in aggressive B-cell lymphoma, if further augmented with an anthracycline.

**Figure 2.**
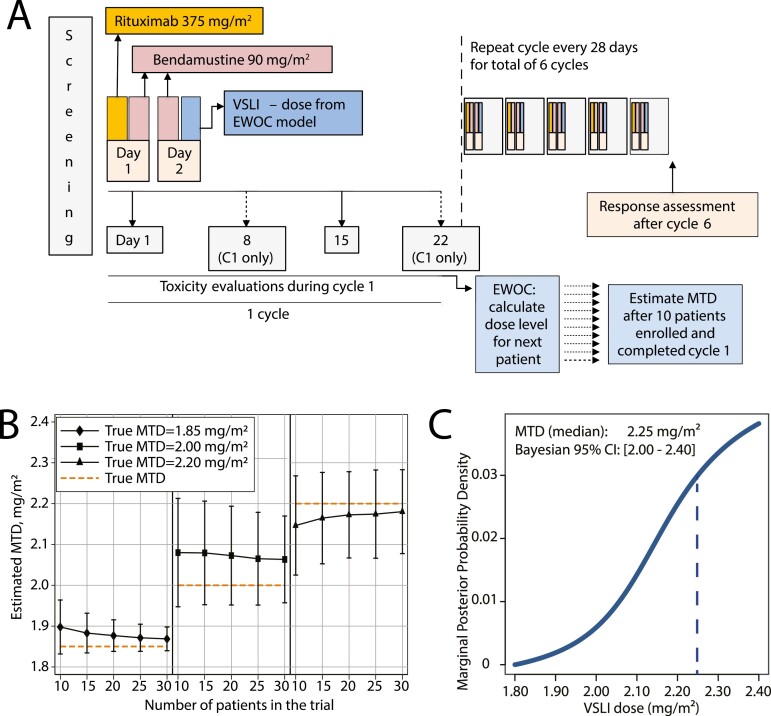
(**A**) Study schema; the EWOC model was used to calculate the VSLI dose for each patients, starting from the first dose of 1.80 mg/m^2^; EWOC input parameters included: target probability of DLT (q) 33%, probability of exceeding target dose (a) 50%, minimal VSLI dose 1.80 mg/m^2^, maximal VSLI dose 2.40 mg/m^2^, cohort size *n* = 1, prior distribution *r*_0_ uniform, *g* uniform; (**B**) EWOC simulations (number of simulated trials: 500) showing estimated MTD and its root mean standard error (whisker bars) against trial sample size, assuming true MTD of 1.85-2.20 mg/m^2^; (**C**) final model-based calculation of the MTD in the current trial; MTD was defined as the median of Bayesian 95% credible interval (CI).

In order to rapidly achieve the MTD of VSLI while maintaining safety, we used the EWOC design, which allows for dose escalation in cohorts of *n* = 1 (Fig.2A) [3, 12]. The Bayesian EWOC model targeted 33% probability of DLT with potential VSLI doses ranging from 1.8 up to 2.3 mg/m^2^. Simulations indicated that with single-patient cohorts, the performance characteristics of the model (MTD estimate and its root standard error) would be adequate even with a sample size of *n* = 10 in the proposed range of VSLI doses (Fig. 2B). Secondary objectives of the trial included cumulative rates of adverse effects (AE), the rate of completion of six cycles of immunochemotherapy, ORR, and CR rate, assessed using the standard computed tomography (CT)-based International Working Group criteria [13, 14]. The study (NCT02257242) was approved by the Institutional Review Board at Rhode Island Hospital and was supported by Acrotech Biopharma, the maker of VSLI.

Although patients with BCL/MCL were eligible for our trial regardless of prior treatments, all enrolled subjects received BR+VSLI as first-line therapy. Among 12 screened patients, one was found ineligible and one could not be treated due to a temporary drug unavailability. The MTD was calculated using experience from the 10 treated subjects (Fig. 2C). However, because only the last two subjects received VSLI at doses >2.20 mg/m^2^, a larger cohort would be necessary to fully characterize the AE profile and efficacy of BR+VSLI at the MTD. We elected not to pursue a dose expansion cohort because of unlikely enrollment success of such an expansion.

One of the factors influencing our decision was the observation of four recurrences with median 27 months of follow-up. Admittedly, two recurrences were seen in mantle cell lymphoma (including one blastoid mantle cell lymphoma) and one in SLL—histologies in which BR is less impressive, with median PFS of 3-4 years [1, 6, 15, 16]. The ORR (100%) and CR rate (50%) in this study were similar to prior experience with BR: in the phase III trial study by Rummel et al. the ORR and CR rates were 93% and 40%, respectively, and in the phase II trial by Flinn et al. they were 97% and 31%, respectively [1, 17]. Of note, the CR rates are higher when measured using combined positron emission tomography (PET) and CT criteria, which we did not use to facilitate comparability with other trials [18].

We noted a higher rate of alopecia (40%, all G1) than expected with BR alone. Peripheral sensory neuropathy was common (50%), but G1/G2 only, and led to early discontinuation of VSLI in only one patient. No dose reductions of bendamustine were required. We observed a significant and protracted lymphopenia after BR+VSLI—of significance particularly in the context of the emerging COVID-19 pandemic. Prolonged lymphopenia (ALC <0.8 × 10^9^/L) occurred in most patients, and 2 patients age > 60 had ALC <0.5 × 10^9^/L beyond 1 year from therapy. BR alone is known to be associated with increased risk of late infections, including pneumonia and opportunistic infections (herpes zoster, cytomegalovirus viremia), especially when followed by maintenance rituximab [18-20]. We observed abnormally low CD4 counts (0.19-0.40 × 10^9^/L) in 5 studied subjects at 18 to 24 months after treatment. Only one patient in our study received maintenance rituximab; this patient had FL and attained partial response with study therapy. No opportunistic infections were recorded, and prophylactic antimicrobial therapy was not mandated.

Recent data suggest high activity of immunomodulatory agents like lenalidomide [21]. targeted agents like B-cell receptor inhibitors [15, 22], and novel immunotherapies including bispecific antibodies for treatment of B-cell lymphoma and mantle cell lymphoma [23]. Therefore, considering the overall toxicity and efficacy results from this study, we concluded that chemotherapy-free first-line approaches may hold more benefit for patients with indolent B-cell lymphoma and mantle cell lymphoma than intensified cytotoxic chemotherapy, and we initiated a trial using such an approach (NCT04792502) [24].

## Data Availability

The data underlying this article will be shared on reasonable request to the corresponding author.
